# Compactibility and Fibre Volume Fraction Limits of Unidirectional Discontinuous Carbon Fibre Thermoset Prepreg Laminates

**DOI:** 10.3390/polym18121472

**Published:** 2026-06-12

**Authors:** Miriam Preinfalck, Julian Kupski, Mohammad Hajikazemi, Christian Brauner, Stephan Baz, Götz T. Gresser

**Affiliations:** 1 Deutsche Institute für Textil- und Faserforschung, Körschtalstraße 26, 73770 Denkendorf, Germany; stephan.baz@ditf.de (S.B.); goetz.gresser@ditf.de (G.T.G.); 2 Institute of Polymer Engineering, FHNW University of Applied Sciences and Arts Northwestern Switzerland, Klosterzelgstrasse 2, 5210 Windisch, Switzerland; julian.kupski@fhnw.ch (J.K.); mohammad.hajikazemi@fhnw.ch (M.H.); christian.brauner@fhnw.ch (C.B.)

**Keywords:** staple carbon fibre, fibre orientation distribution, ply compactibility, unidirectional laminates, fibre volume content, thermoset prepreg, recycled carbon fibre

## Abstract

The aim of this study was to explore the compactibility of unidirectional staple carbon fibre laminates in comparison with their uni- and biaxial continuous fibre counterparts. Resin-preimpregnated plies were inserted into a heated compression mould at an elevated mould temperature of 110 °C. By applying stepwise loading, the correlation between consolidation pressure and fibre volume content was derived and related to fibre orientation distribution. The fibre orientation distribution is obtained from photographic image analyses of 2D ply sections of the same samples using the structure tensor approach. For commonly used autoclave prepreg pressure of 6.8 bar results indicate that lower-oriented staple carbon fibre unidirectional laminates with a fibre orientation distribution factor *η*_0_ = 0.74 can potentially reach a maximum of 39% fibre volume fraction, while higher-oriented laminates with *η*_0_ = 0.78 end up at 43%. An exponential extrapolation suggests that a consolidation pressure of ≥90 bar is required to achieve 60% fibre volume content with highly oriented unidirectional staple carbon fibre laminates.

## 1. Introduction

Carbon fibre reinforced polymers (CFRPs) enable outstanding specific stiffness and strength and are therefore widely used in lightweight structural applications. However, increasing production volumes and limited recyclability of thermoset matrices have intensified the demand for resource-efficient material solutions. The reuse of carbon fibres, particularly in the form of short, recycled staple carbon fibres (CF), has thus emerged as a promising strategy to reduce environmental impact while retaining high mechanical performance [1].

Staple fibre composites consist of discontinuous fibres typically ranging from millimetres to centimetres in length. Compared with continuous fibre systems, they offer broad material utilisation, lower cost, and greater flexibility in processing [2,3]. However, fibre discontinuity introduces microstructural heterogeneity, fibre-end gaps, and imperfect alignment, all of which influence mechanical performance and consolidation behaviour.

Carding is commonly employed to align staple fibres prior to tape or yarn formation. During this process, fibres are carded (oriented by means of metal pins, similar to brushing), aligned, and formed into a uniform web, which is then condensed to slivers and subsequently into yarn or tapes for composite manufacturing [4,5,6,7]. The resulting fibre architecture governs both the load transfer efficiency and the packing behaviour during consolidation. Consequently, controlling fibre orientation distribution (FOD) is essential for achieving structural-grade performance [8,9].

Besides the material properties of the constituents and the fibre-matrix interface properties [10,11], the mechanical response of fibre-reinforced composites is determined by microstructural descriptors such as fibre orientation [12,13,14,15], fibre length [16,17], interfibre spacing [18], fibre diameter [19], fibre connectivity and curvature [20], fibre volume content (FVC) [21], and porosity [22,23]. These features are quantified using destructive methods (optical microscopy [24,25], mechanical testing [7,26,27]) and non-destructive techniques including X-ray diffraction [28], X-ray computed tomography [18,20], ultrasound-based methods [29,30], and spectroscopic approaches [31]. Image analysis techniques such as Fourier transforms and structure tensor methods enable statistical evaluation of FODs [15,32]. Moreover, micromechanical models frequently employ modified rule-of-mixtures formulations to relate microstructure to elastic properties. However, such models typically assume a known and uniform FVC. In staple fibre laminates, the attainable FVC is itself a function of fibre architecture and consolidation pressure, introducing a coupling between microstructure and process mechanics that is often neglected.

In unidirectional (UD) continuous fibre laminates, fibres are ideally aligned and assumed infinitely long. In contrast, staple fibre laminates exhibit both finite fibre length and a distribution of fibre orientation angles [20]. These effects can be incorporated into the longitudinal modulus prediction using efficiency factors. These efficiency factors were implemented for oriented staple fibre architecture laminae [24,33,34]:

(1) $$E_{11}=\eta_{0}\eta_{1}V_{f}E_{f}+V_{m}E_{m}$$

where $E_{11}$ is Young’s modulus of the composite; $\eta_{0}$ is a correction factor for the fibre orientation; $\eta_{1}$ is a correction factor accounting for the overlap length between adjacent fibres, based on the Cox model [35]; $V_{f}$ and $E_{f}$ are the fibre properties; and $V_{m}$ and $E_{m}$ are the matrix properties. $\eta_{0}$ is defined as

(2) $$\eta_{0}=\sum \beta_{i}{cos}^{4}\theta_{i}$$

with $\beta_{i}$ denoting the frequency of fibres oriented at angle $\theta_{i}$. For perfectly aligned fibres, $\eta_{0}=1$. For fibre lengths significantly exceeding the critical transfer length (≫1 mm in typical carbon/epoxy systems), $\eta_{1}\approx 1$, indicating that stiffness reduction is dominated by orientation rather than shear-lag effects. From a tensorial perspective, $\eta_{0}$ corresponds to a scalar representation of the second-order orientation tensor projected onto the loading direction. Beyond stiffness reduction, fibre misalignment also affects packing density. Increasing angular deviation increases excluded volume and limits fibre nesting during compaction. Thus, orientation distribution simultaneously governs mechanical efficiency and achievable fibre volume fraction.

The compaction of fibre beds can be described as a pressure-dependent reduction in void space within a granular-like network of interacting filaments or fibres. For discontinuous fibres, three mechanisms dominate:

(i) Fibre reorientation and nesting at low pressures;
(ii) Bending and local rearrangement at intermediate pressures;
(iii) Fibre deformation (up to fibre breakage) and matrix-dominated compression at high pressures.

Staple fibre preforms are generally more voluminous than continuous fibre laminates due to fibre-end gaps, waviness (undulation and crimp), degree of fibre orientation and imperfect stacking. Even in theoretically aligned systems, fibre ends introduce unavoidable geometric inefficiencies. The shorter the fibres, the higher the frequency of end gaps and the lower the maximum attainable packing density [36]. From practical manufacturing experience, achieving FVC ≈ 60% under autoclave pressures of 6–7 bar is challenging for staple fibre laminates. Misaligned fibres increase steric hindrance and cause premature densification plateaus, requiring substantially higher consolidation pressures compared with continuous fibre systems.

Edwards and Evans [36] first quantified the pressure–volume relationship of aligned short fibre mats. Centrifugally aligned 3 mm CF mats achieved FVC ≈ 50% at 7 bar and ~60% at 69 bar. While continuous fibre mats approached ~70% FVC, discontinuous systems were theoretically limited to ~47% at low pressure due to fibre-end effects, highlighting intrinsic packing constraints. Eom et al. [37] linked fibre bed compressibility to cure-induced stress, identifying a consolidation window of 1–5 bar for achieving 50–62% FVC without void formation. Likewise, Kirupanantham [34] reported non-linear densification of discontinuous carbon fibre laminates, with compaction plateauing above 20 bar and final FVC values of ~55–58%, indicating structural packing limits rather than insufficient pressure. Subsequent studies focused on improving the understanding and characterisation of fibre bed compaction. Experimental and modelling approaches demonstrated reliable prediction of laminate densification [38], while investigations of bundle mechanics and packing behaviour highlighted the influence of fibre architecture, needling, and sizing on compaction response and moulding performance [39,40]. At the same time, work on testing methodology addressed inter-laboratory variability, machine compliance, and in situ characterisation to improve the accuracy and process relevance of compaction measurements [41,42,43].

Although the compaction behaviour of textile reinforcements and continuous fibre systems is well established, quantitative relationships between FOD and achievable FVC in staple CF prepregs remain unresolved. Existing studies typically characterise material properties at comparatively low FVC and extrapolate to higher compaction levels, despite limited evidence that such FVCs are attainable under realistic consolidation pressures. A predictive framework linking *η*_0_, consolidation pressure, and resulting FVC under industrially relevant autoclave conditions is currently absent. Resolving this gap is critical for defining manufacturable design windows and for assessing whether improvements in fibre alignment can offset pressure limitations in industrial processing. Accordingly, this study quantifies for the first time the compactibility of UD staple CF thermoset prepreg laminates relative to continuous fibre systems and establishes a mechanistic relationship between FOD and achievable FVC as a function of consolidation pressure.

## 2. Materials and Methods

### 2.1. Materials

**Figure 1:**
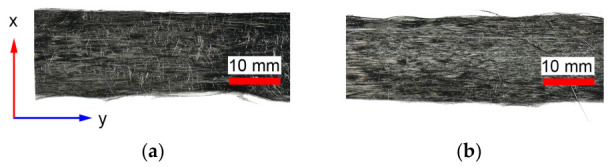
Top-view images of the tapes utilised in this study depicting: (**a**) low-stretch (LS) and (**b**) high-stretch (HS) tape, both with a nominal tape width of 16 mm [15].

The methodology was demonstrated using two types of in-house manufactured UD staple CF tapes with varying alignment processing parameters, designated as low-stretch (LS) and high-stretch (HS). The average length of a single fibre in these tapes was approximately 50 mm, and the average fibre diameter was 7 μm. The fibre used in all samples was of the standard modulus/high-strength type, T700SC-12K-50C, following the naming convention of Toray Industries, Inc. (Tokyo, Japan). Alongside the staple CF tapes, two reference samples were produced using the same fibres in a dry UD carbon layup with 80 g/m^2^. Table 1 depicts an overview of the layup and the areal weight of all configurations, taking into account the sizing amount on the chosen fibre and a small deviation in added binder between staple and continuous fibre preforms. Figure 1 shows optical images of both staple fibre tape configurations, HS and LS, utilised in this study.

A total of 12 layers of staple CF tapes, as well as reference continuous CF tapes of 83.5 g/m^2^ areal weight, were laminated to form flat test specimens. Resin films with an areal weight of 100 g/m^2^ were produced using the HLCL-1000 hot melt laboratory coater from ChemInstruments (Fairfield, OH, USA). These resin films were then used to manufacture prepregs by film-stacking them with the tapes in the desired layup configuration. To prevent excessively rapid curing of the resin, only the EPIKOTE™ Resin 05545 (Westlake Corporation, Houston, TX, USA) component was employed as a film to the dry tapes. Consequently, potential sources of variability associated with curing reactions, such as mixing ratio deviations, pot-life effects, or exothermic heat generation, were excluded. A preliminary rule-of-mixtures calculation indicates that the areal weight of the supplied resin film would exceed the amount typically required to achieve FVC in the range of 50–60%. This was intentionally selected to ensure an adequate resin supply during testing and to minimise the risk of void formation. The tapes were carefully aligned in parallel and accurately cut to match the dimensions of the mould cavity. Prior to each testing cycle, the mould cavity and stamp were coated with Zyvax^®^ WaterShield™ EU (Chem-Trend, Howell, MI, USA) as a release agent.

### 2.2. Mesoscopic FOD Analysis Method

In this study, the FOD analysis technique based on photographic imaging of each ply of the laminate was chosen. This method was previously introduced by Zweifel [15], and the present work aims to link his past FOD visual inspection results with novel compaction behaviour curves for the same samples. Image analysis based on 2D ply sections was performed using the structure tensor approach to characterise FODs. The structure tensor is a matrix that describes the local second-order structure of an image and is often used in computer vision and image processing for edge detection, texture analysis and directionality. Furthermore, the degree of homogenisation or representative volume element (RVE) was addressed in the frame of different region of interest (ROI) volumes.

FODs were determined using a two-dimensional structure tensor approach implemented in Python (v3.8.12). During plate manufacturing, each ply was photographed with a calibrated DSLR setup (Nikon D810, Nikon Corporation, Tokyo, Japan) equipped with a 50 mm lens and a circular polarisation filter to minimise reflection artefacts. Images (5′759 × 2′879 pixels, 300 dpi) were corrected for lens distortion using OpenCV [13]. Automated image processing subdivided the images into block segments for spatial homogenisation. Fibre orientations were extracted via a convolution-based structure tensor method and orientation histograms were converted into probability density functions. The alignment coefficient $\eta_{0}$ was calculated for each segment. Virtual stacking of the plies yielded laminate-level median and standard deviation values of $\eta_{0}$ for each configuration. Using this method, it was revealed that most fibres that are not correctly aligned appear at the ply surface or boundaries. Thus, 2D mesoscopic analysis can be utilised to identify critical fuzzy regions in the tape.

### 2.3. Specimen Manufacturing and Testing

A square consolidation mould with a 32 × 32 mm^2^ cavity was developed for integration into a universal testing machine (K100, Zwick Roell GmbH & Co. KG, Ulm, Germany), enabling controlled stepwise hydrostatic compaction and precise thickness measurement at elevated temperature [44]. Tests were conducted at 110 °C. This processing temperature was defined according to Walker [45], who identified 110 °C as optimal for binder dissolution from the staple CF tapes into the epoxy matrix within several minutes. At lower temperatures dissolution remains incomplete, whereas at higher temperatures resin curing may outpace binder diffusion, potentially influencing consolidation behaviour.

**Figure 2:**
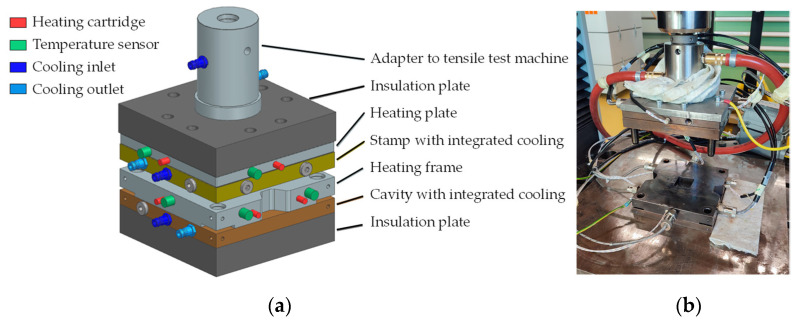
Consolidation experiment setup [44]: (**a**) schematic illustration; (**b**) photograph of the setup installed in the testing machine.

The lower mould assembly consisted of a heated frame mounted on a water-cooled base plate and supported by a rigid steel backing plate to minimise compliance. The upper assembly comprised a water-cooled stamp with integrated heating, connected to the load cell via a cooled adapter (Figure 2).

Machine compliance can significantly affect compaction measurements if not properly corrected, as demonstrated by Sousa et al. [42]. In the present setup, ceramic insulation plates were identified as the primary source of compliance and were therefore removed. Thermal control was ensured through enhanced active cooling of the upper mould and the addition of a rigid base plate to moderate heat transfer (Figure 2). Residual system deformation was quantified by performing empty mould runs, which were subtracted from the measured specimen displacement. All tests were executed after passing a 30 min heat-up of the entire test setup to ensure thermal equilibrium and avoid displacement artefacts caused by transient thermal expansion of the tooling.

**Figure 3:**
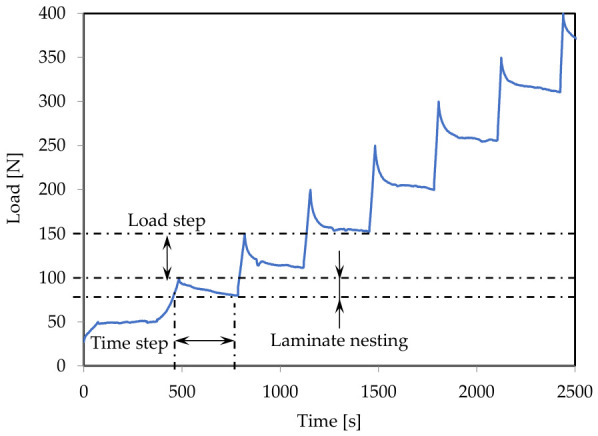
Extract from the raw data curve: load over time with load reduction after 300 s at constant displacement.

In accordance with Kirupanantham [34], compaction was treated as a time-dependent process governed by resin flow and viscoelastic relaxation. Under constant load, laminate thickness continued to decrease due to resin redistribution within the fibre network, particularly at low consolidation pressures. The load was applied incrementally at a crosshead speed of 0.2 mm/min in 50 N steps, from an initial load of 50 N up to 2000 N (40 load steps). The selected number of load increments represents a compromise between achieving sufficient data resolution for deriving pressure–FVC correlation curves and limiting overall test duration. Prolonged testing may lead to gradual thermal drift of the moulding setup despite the integrated cooling system, potentially affecting calibration and displacement accuracy. At each level, displacement was held constant for at least 300 s to allow force relaxation and to ensure quasi-equilibrium thickness before proceeding to the next increment. The resulting load over time/displacement data, as displayed in Figure 3, were used to derive correlation curves linking consolidation pressure to achievable FVC. The FVC was thereby calculated using the following expressions:

(3) $$FVC=\frac{V_{f}}{V}\;\;\;\;\;\;\;\;\;\;\;\;\;\;\;V_{f}=\frac{m_{f}}{\rho_{f}}\;\;\;\;\;\;\;\;\;\;\;\;\;\;\;V=A\times \Delta h$$

with Δh corresponding to the measured displacement of the testing machine, A being the mould surface area, and $\rho_{f}$ = 1.79 g/cm^3^, representing the CF density.

The gap between the upper and lower mould enabled excess low-viscosity epoxy to flow out of the cavity during processing. Post-test observations show significant resin accumulation outside the cavity, consistent with the initially supplied excess resin, indicating that sufficient drainage occurs and that the measured thickness is primarily governed by the fibre preform.

## 3. Results

### 3.1. FOD Analysis Results

**Figure 4:**
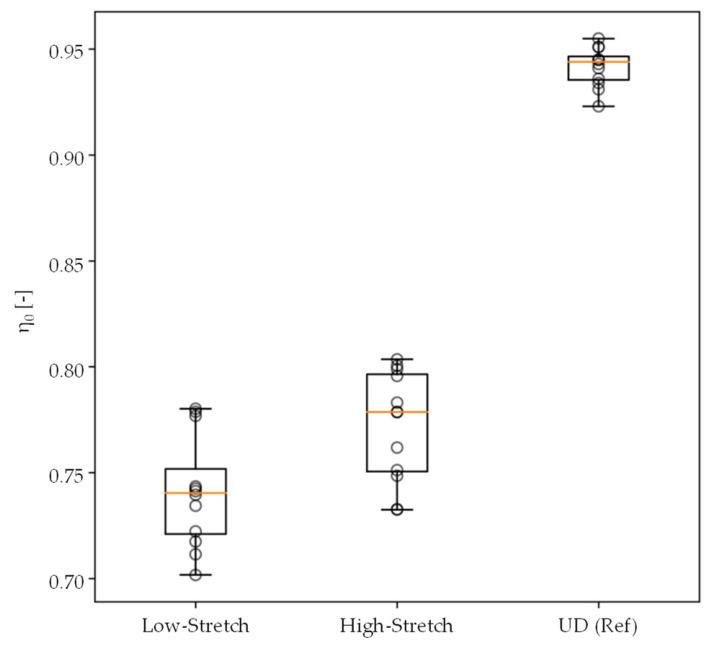
Derived $\eta_{0}$ values from virtually stacked optical images acquired ply-by-ply with a Digital Single-Lens Reflex (DLSR) camera.

Figure 4 presents the results of the FOD analysis. Zweifel [15] measured $\eta_{0}=0.741$ for the LS and 0.781 for the HS tape configuration. In comparison, a UD continuous CF non-crimped fabric was measured with $\eta_{0}=0.934$. These measurements were taken on dry preforms prior to consolidation.

### 3.2. Derivation of Pressure over FVC Curves

**Figure 5:**
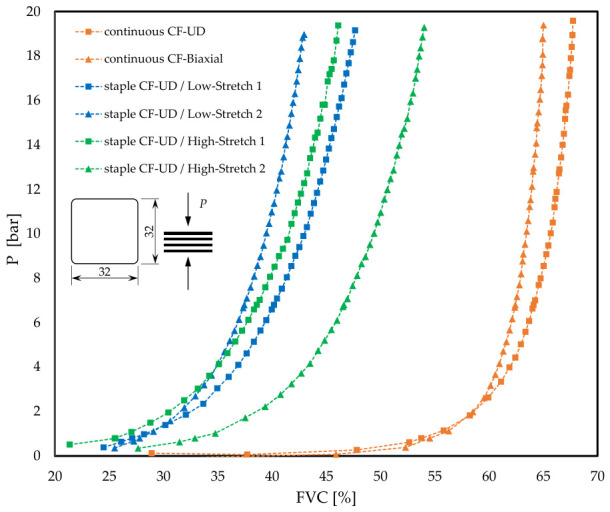
Cavity pressure over FVC for UD staple and continuous CF tapes.

The calculated compaction curves relating mould pressure to FVC are presented in Figure 5. Continuous CF laminates exhibit significantly higher compactibility under moderate pressure compared with staple CF systems, even for biaxial layups. In continuous UD and biaxial configurations, efficient intra-ply filament nesting enables rapid densification at low pressures. In contrast, UD staple CF laminates display reduced packing efficiency due to fibre discontinuity and misalignment. Notably, substantial differences were observed between staple CF specimens manufactured from the same base material. The HS configuration exhibited increased scatter in the pressure–FVC correlation.

The observed behaviour is consistent with trends reported by Kirupanantham [34] and Grieder [44], showing non-linear densification with a pronounced plateau at higher pressures.

**Figure 6:**
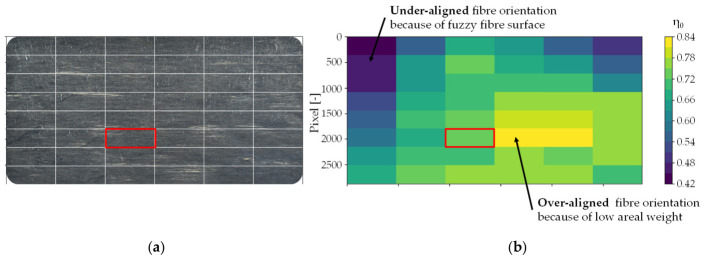
Over- and under-alignment regions on scanned layer surfaces. (**a**) photograph of a ply using a polarisation filter; (**b**) false-colour fibre orientation map.

This variability is attributed to regional FOD differences and local thickness variations, as previously indicated by Zweifel [15], see Figure 6. There, the most representative segment was chosen by fitting a Weibull distribution to the apparent fibre orientation correction factor derived from histograms (structure tensor). A Weibull distribution was chosen because the average value does not represent the data well.

These inhomogeneities originate from the carding and the subsequent alignment process of the staple fibres to tapes as well as from the alignment of the tapes. Variations in the initial fibre length distribution extend beyond the well-characterised profiles produced by modern processing machinery [2,3]. These fibre length variations contribute to non-uniform sliver formation, leading to localized regions enriched in either shorter or longer fibres. During subsequent alignment, due to the big difference between the shortest and longest fibres, the uniformity variations intensify, leading to areas of increased fibre-end density and locally thicker tape sections, alongside regions dominated by longer fibres that appear comparatively thinner. Such spatial heterogeneity directly affects compaction response and contributes to the observed scatter in FVC.

### 3.3. Data Extension to Distinct Process Parameters

**Figure 7:**
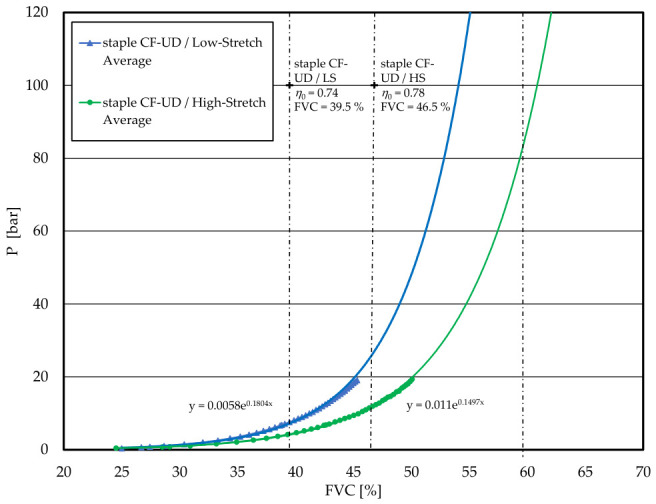
Averaged pressure–FVC curves for LS and HS configurations, exponentially extrapolated.

Linear interpolation and exponential extrapolation of the compaction curves were performed to provide an indicative estimate of FVC under typical processing conditions, presented in Figure 7 and Table 2. The extrapolation was done without the introduction of formal uncertainty bounds. For vacuum processing (0.8 bar), predicted FVC values reach 27.4% (LS) and 29.1% (HS). Under autoclave conditions (6.8 bar), FVC increases to 38.8% (LS) and 42.6% (HS).

For the HS configuration, achieving an aerospace-grade FVC of approximately 60% appears to require pressure levels on the order of ~90 bar. Hereby, two visually distinct parts of the curves in Figure 7 represent the two different data types: The first segment (lines with markers) corresponds to the experimentally measured compaction data consisting of 40 discrete load steps. The second segment (continuation of lines without markers) represents the exponential fit applied to the experimental data.

However, this estimate should be interpreted cautiously, since it extends beyond the experimentally investigated pressure range and is influenced by experimental scatter and local laminate heterogeneity. For the LS configuration, the required pressure would theoretically exceed this range even more substantially. The extrapolation serves primarily to illustrate the practical limitations of pressure-driven densification in the investigated staple CF system rather than to provide an exact predictive threshold.

**Figure 8:**
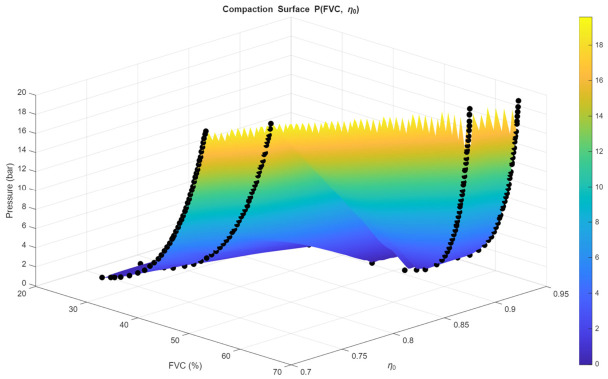
Surface showing the required compaction pressure as a function of FVC and fibre orientation factor (*η*_0_) for staple CF laminates.

Another 60 under reasonable autoclave processing conditions (~6 bar). To obtain this information, the data in Figure 5 were combined with FOD information expressed in terms of *η*_0_ and a surface was fitted to obtain an interpolated function of the pressure required as a function of FVC and *η*_0_. For this curve to be highly precise, a large number of compaction tests would likely be needed for staple CF composites with different FOD levels, which does not seem feasible. Additionally, the same tape was used to manufacture continuous fibre laminates in both unidirectional and biaxial layups, resulting in similar *η*_0_ values for these two laminate types. Some scatter is observed in tests for both low-stretch and high-stretch samples, even when using a single *η*_0_ to express their in-plane FOD. However, to gain a practical understanding of how FOD affects the achievement of aerospace-grade FVC in realistic autoclave processing, the FVC–pressure results of different staple CF laminates were averaged in Figure 7. A slightly lower value of *η*_0_ = 0.9 was assigned to the biaxial laminate, and a surface was fitted to express pressure as a function of FVC and *η*_0_. The resulting surface is shown in Figure 8. Analysis of this figure indicates that, for UD staple CF tapes to reach an FVC of 60% at 5.84 bar, *η*_0_ should be approximately 0.89. This is not an absolutely accurate threshold due to the simplifying assumptions made in constructing Figure 8, but it provides useful guidance regarding the effort required to use staple fibres in structural applications.

Finally, it should be noted that the FOD considered in this study represents the in-plane fibre orientation derived from 2D image analysis. The compactibility may also be influenced by additional microstructural features that are not fully captured by this metric, such as out-of-plane fibre orientations, fibre undulations, non-uniform fibre distribution, and resin-rich regions. These characteristics can further reduce packing efficiency, particularly during the initial stage of compaction often described as the fibre reorientation and nesting phase at low pressures. In this regime, the laminate structure behaves more similarly to a loosely stacked multi-ply system, where limited filament mobility and hindered intra-ply nesting restrict early densification.

### 3.4. Comparing FVC-FOD Results with Previous Work

Table 3 compares the present results with previously reported data for UD staple CF laminates. Considerable scatter is observed between studies, both in reported alignment coefficients $\eta_{0}$ and achieved FVC values. For example, HiPerDiF systems [22] with fibre lengths of 1–3 mm achieved $\eta_{0}\;$ ≈ 0.90–0.92 and FVC values between 41 and 55%, while the TuFF process [27] reported FVC up to 63% at similar orientation levels. Given 100 bar of consolidation pressure, Walker [45] reported FVC ≈ 53% at $\eta_{0}=0.85\;$ for 50 mm fibres. With the same processing parameters Zweifel [15] measured lower alignment factors for the same 50 mm staple fibre tapes ($\eta_{0}=0.741$ for LS and 0.781 for HS), corresponding to FVC values of 39.5% and 46.5%, respectively.

The deviation between extrapolated predictions and experimentally obtained FVC values can largely be attributed to spatial FOD inhomogeneities. Over- and under-aligned regions reduce the effective laminate-level $\eta_{0}$, limiting achievable packing density despite local alignment improvements. Furthermore, direct comparison between studies is complicated by differences in FOD and FVC measurement techniques, as well as variations in fibre length, diameter and surface topology. Zweifel assumed a fictitious fibre length of 10 mm within his structure tensor approach. Experimental measurements after the carding process, however, indicated approximately 50 mm average length. Although fibre length primarily affects the shear-lag efficiency factor $\eta_{1}$ rather than $\eta_{0}$, it may still influence packing behaviour and thus the attainable FVC through fibre-end density and nesting characteristics. The comparison between studies should therefore be interpreted with caution. Besides differences in FOD and FVC measurement methodologies, variations in fibre length, diameter, surface topology, and processing routes may influence the reported compaction behaviour. In the present work, fibre length was not systematically varied and therefore its individual contribution to compactibility and attainable FVC cannot be isolated from the effects of fibre orientation and regional heterogeneity. Although fibre length may influence packing behaviour through fibre-end density and nesting characteristics, the current dataset does not allow quantitative conclusions regarding this effect. Accordingly, potential fibre-length influences should be regarded as a limitation of the present study and as a subject for future investigation rather than as a demonstrated mechanism.

## 4. Conclusions

Compaction trials were conducted to determine the maximum practical FVC of UD staple CF thermoset prepreg tapes under varying hydrostatic pressures. Square specimens of 32 × 32 mm^2^ were manufactured from two alignment states (LS and HS) and compared with continuous CF [0]_12_ and biaxial [0/90]_3S_ laminates. Pressure–thickness data were evaluated to derive correlation curves linking consolidation pressure to achievable FVC. These results were subsequently linked to FOD mesoscopic image analysis for the same UD staple CF tapes in dry state.

The following conclusions can be drawn:

▪ Continuous CF laminates exhibit significantly higher compactibility under moderate pressures compared to UD staple CF tapes. Efficient intra-ply filament nesting enables rapid densification, even for biaxial stackings, whereas fibre discontinuity and misalignment limit packing efficiency in staple systems.
▪ The compaction response of staple CF laminates is strongly governed by FOD. Both mesoscopic image analysis and mechanical compaction trials indicate that higher nominal alignment (HS) is associated with increased scatter in FVC. This behaviour is attributed to spatial inhomogeneities introduced during the carding and alignment process, leading to locally over- and under-aligned regions.
▪ Extrapolation of the compaction curves demonstrates that aerospace-grade FVC values (~60%) would require consolidation pressures approaching 90 bar for the investigated material system. Such pressure levels exceed typical autoclave capabilities, indicating that pressure increase alone is not a viable route for achieving structural-grade FVC in current UD staple CF prepregs.

A limitation of the present study is that fibre length was not independently varied. Consequently, while fibre orientation effects on compactibility could be evaluated, the individual influence of fibre length and fibre-length heterogeneity on achievable FVC could not be isolated and therefore should not be interpreted quantitatively from the present dataset. Future work should therefore focus on optimising the carding and alignment processes, as well as exploring strategies to enhance staple CF alignment during part shaping, with the aim of reducing regional FOD variations and identifying an optimal balance between fibre length, alignment quality, and packing efficiency.

## Figures and Tables

**Table 1:** Laminate properties of tested coupons with Torayca T700SC 12K-50C.

	Layup	Binder + Sizing Portion [m%]	Dry Areal Weight [g/m^2^]
UD staple CF—LS	[0]_12_	11.05	80
UD staple CF—HS	[0]_12_	11.05	80
UD continuous CF	[0]_12_	5.34	83.5
Bi-axial continuous CF	[0, 90]_3s_	5.34	83.5

**Table 2:** Inter- and extrapolated FVC for typical prepreg consolidation processes.

	P [bar]	FVC [%]
Vacuum processing/LS	0.8	27.4
Autoclave processing/LS	6.8	38.8
Aerospace grade/LS	n/a	60.0
Vacuum processing/HS	0.8	29.1
Autoclave processing/HS	6.8	42.6
Aerospace grade/HS	~90	60.0

**Table 3:** Comparison with FOD/FVC values from the literature.

	Average Fibre Length [mm]	*η*_0_ [-]	FVC [%]
Yu, HiPerDiF [24]	1	0.90	41
Yu, HiPerDiF [24]	3	0.92	55
Yarlagadda, TuFF [27]	5	0.91	63
Walker, FHNW [45]	50	0.85	53
UD staple CF—LS [15]	10/50	0.74	40
UD staple CF—HS [15]	10/50	0.78	47

## Data Availability

The original contributions presented in this study are included in the article. Further inquiries can be directed to the corresponding authors.
